# Exploring the prognostic value of the neutrophil-to-lymphocyte ratio in cancer

**DOI:** 10.1038/s41598-019-56218-z

**Published:** 2019-12-23

**Authors:** Rachel Howard, Peter A. Kanetsky, Kathleen M. Egan

**Affiliations:** Department of Cancer Epidemiology H. Lee Moffitt Cancer Center and Research Institute, Tampa, FL USA

**Keywords:** Cancer epidemiology, Prognostic markers

## Abstract

In cancer patients, a high pre-treatment neutrophil-to-lymphocyte ratio (NLR) is associated with poorer survival outcomes. Significant variation in the magnitude of this association has been observed between studies, but sources of this variation are poorly understood. Here, we explore differences in the prognostic potential of NLR between patient subgroups stratified by demographic and clinical characteristics using a retrospective cohort of 5,363 patients treated at Moffitt Cancer Center (Tampa, FL). We identify patients for whom NLR has maximum prognostic potential via adjusted hazard ratios (HRs) calculated using multivariable Cox proportional hazards models and area under the curve analysis. NLR demonstrates stronger associations (HRs > 2) with survival among African-American patients, patients receiving radiation therapy, stage IV patients, and melanoma patients when compared with the overall study population (HR = 1.58). Sensitivity and specificity of NLR as a prognostic marker are also higher in these patient subgroups, and increase further with combinations of multiple “high-risk” demographic or clinical characteristics. In summary, NLR may have greater prognostic value in patients with certain demographic and clinical features. Future prospective studies could validate this hypothesis, after further characterization of populations in which NLR has maximum prognostic potential and the identification of meaningful thresholds for risk stratification.

## Introduction

Inflammation plays a key role in the pathophysiology of many diseases, including cancer^1–4^, and identifying easily obtainable and robust metrics for the status of systemic inflammation has become a high priority. In cancer, such metrics could serve as prognostic biomarkers, permitting risk stratification at diagnosis and offering the prospect of patient matching to specific therapeutic interventions. Immune cell components of the complete blood count (CBC) offer a particularly attractive measure of inflammation as the CBC is often collected as part of standard clinical care at minimal cost and inconvenience to the patient. These CBC components harbor translational potential that is not yet being utilized.

One marker of systemic inflammation available from the CBC is the neutrophil to lymphocyte ratio (NLR), the quotient of the absolute neutrophil and lymphocyte counts^5–8^. Neutrophilia is a common feature of cancer-associated chronic inflammation; although neutrophils are an integral part of the innate immune response, both tumor-promoting and immune-suppressive roles of neutrophil subpopulations have been documented^9–13^. In addition to producing cytokines associated with tumor progression, neutrophils can suppress the activity of cytotoxic T cells and in turn promote metastasis^14,15^. Neutrophilia is commonly accompanied by relative lymphocytopenia, representing a significant decline in the cell-mediated adaptive immune response. The NLR captures the balance between the detrimental effects of neutrophilia and the beneficial effects of lymphocyte-mediated adaptive immunity^16^.

Both systemic neutrophilia and lymphopenia are associated with poorer prognosis in cancer patients^17–20^. Many previous studies have examined the prognostic value of pre-treatment NLR^5,6^, and strong associations have consistently been demonstrated between high NLR and poor patient outcomes across many cancer types. Despite this, significant variation in the strength of association between NLR and survival outcomes has been observed^6,21^. In a meta-analysis, Templeton *et al*. identified significant differences in pooled effect estimates when stratifying studies by cancer type and metastatic versus non-metastatic disease, suggesting the prognostic potential of the NLR may not be equal among all patient subgroups^6^. Specifically, pancreatic cancer, renal cell carcinoma, and mesothelioma demonstrated significantly stronger associations of the NLR with overall survival^6^. The magnitude of NLR also varies by demographic characteristics including age and race^22,23^, yet it remains unclear whether the strength of association with clinical outcomes also varies between these groups.

Here, we conduct an in-depth investigation into the sources of heterogeneity in NLR and its association with patient survival, considering not only cancer type, but also a range of clinical and demographic patient characteristics on which information is routinely collected. We assess individual-level data from a large retrospective cohort of 5,363 patients across 8 cancer sites receiving first line therapy at a leading cancer treatment center in Tampa, FL, to establish average baseline NLR and associations with survival outcomes within demographically and clinically homogeneous patient subgroups.

Despite evidence of strong prognostic potential, uncertainty in the optimal cut-off value defining high-risk NLR and limited understanding of the strength of association between NLR and clinical outcomes in specific patient subgroups are among the barriers to the adoption of the NLR as a tool for prognosis or clinical decision making. It is hoped that by identifying patients for whom the NLR harbors maximum prognostic potential and suggesting corresponding group-specific cut-offs for high NLR, the present study may facilitate the prospective clinical validation of this valuable biomarker as a tool for risk stratification.

## Methods

### Data sources and extraction

The inclusion criteria for the present study were as follows: diagnosis with a solid tumor of the breast, pancreas, liver, esophagus, colon/rectum, prostate, ovary, or skin (melanoma) between 1986 and 2014; receipt of first line treatment at Moffitt Cancer Center; availability of neutrophil and lymphocyte counts within 30 days prior to treatment; and availability of data on primary site, date of diagnosis, date of first treatment, date of last contact or death, and date of neutrophil/lymphocyte count collection. All other patients were excluded from the analytic cohort. An extensive meta-analysis was conducted to complement the present study, featuring 228 published studies and over 75,000 patients^24^. The 8 cancer types prioritized herein were selected based on pooled hazard ratios across the existing literature of above 1.70, and the availability of at least 200 Moffitt Cancer Center patients for inclusion in the study.

The Moffitt Health Research and Informatics (HRI) Data Warehouse was mined to identify patients with blood laboratory results available from prior to first treatment from which the absolute count of neutrophils and lymphocytes could be obtained. The Moffitt HRI Data Warehouse includes clinical, patient demographic and treatment information, as well as dates of diagnosis and last known survival or death in order to compute survival times. HRI data was abstracted and neutrophil and lymphocyte values were obtained from the internal Data Management and Integration (DMIT) group at Moffitt Cancer Center, and additional tumor characteristics and demographic data including gender and race were obtained and verified using the Cerner Powerchart electronic medical records system. All patient data was obtained in accordance with Protocol 18349 (approved by Advarra IRB# 00000971 on 11/12/2018, Pro00014707 Chesapeake, PI: Kanetsky, PA). This was a retrospective study requiring no new data collection, and thus a waiver of HIPAA authorization and consent was requested and approved from the IRB. All protected health information was de-identified by an independent honest broker in the Collaborative Data Services Core service at Moffitt Cancer Center before being provided to the investigators for analysis.

Clinical and demographic variables previously shown to harbor prognostic potential in cancer patients and that were available across all cancer sites of interest were selected as covariates. These included age at diagnosis (<60, ≥60; cut-off for age selected based on existing literature relating to NLR^25,26^), sex (male, female), race (black, white, other), disease stage (TNM classification; stage I-IV), histology type (well differentiated, moderately differentiated, poorly differentiated, undifferentiated), and treatment regime (surgery, radiation therapy, chemotherapy, immunotherapy). A total of 5,363 patients were included in the final analysis (breast N = 979, colorectal N = 1,024, esophageal N = 594, liver N = 281, melanoma N = 349, ovarian N = 245, pancreatic N = 1,276, prostate N = 615).

### Statistical analysis

#### Summary statistics

Descriptive statistics including median, quartiles and range characterize baseline NLR in subgroups of patients stratified by clinical and demographic characteristics of the patient (cancer type, disease stage, age, sex, race).

#### Variation in NLR

Mann Whitney U tests were performed to identify statistically significant differences in baseline NLR between strata. For variables with more than two strata (cancer type, disease stage, race) Bonferroni-corrected pairwise comparisons were conducted in addition to the Kruskal-Wallis omnibus test for differences between and among strata, respectively.

#### Kaplan Meier analysis

Kaplan-Meier analysis using log-rank testing estimated the difference in overall and disease-specific survival between patients with high and low NLR for the population as a whole, with the cutoff for high NLR defined as the median NLR in the complete cohort. Similar analyses were then conducted comparing survival between patients with high and low NLR within each variable of interest. For example, the separation of high and low NLR survival curves was compared by evaluating the difference in both median survival time and log-rank test p-value between males and females, between cancer types, and so on. To prevent differences in baseline NLR across groups from masking true relationships with survival, cutoffs for high NLR were defined for each respective group based on the median NLR among the patients within that group. Note that for survival and proportional hazards analysis, only patients receiving single-modality treatment were included to avoid confounding by receipt of multiple therapies.

#### Cox proportional hazards analysis

Multivariable Cox proportional hazards models were used to determine adjusted HRs and 95% CIs for NLR and overall and disease-specific survival for the cohort as a whole. The previously mentioned variables (cancer type, disease stage, age, race, sex) as well as disease histology and treatment type were all evaluated in univariate analysis, and significant variables included in the multivariable model. Violation of the proportional hazards assumption was evaluated by calculating Schoenfeld residuals and p-values, and in the case of non-proportionality stratified Cox regression was conducted to evaluate the effect of non-proportionality on the resulting HRs. Log-rank trend tests were conducted to evaluate trends in the resulting hazard ratios where relevant (disease stage, differentiation status). In addition to evaluating the association between NLR and survival in the cohort as a whole, we further calculated adjusted HRs for high NLR and overall and disease-specific survival (all-time, five year, and ten year) within demographically and/or clinically homogeneous patient subgroups. Again, group-specific cutoffs for high NLR were defined for each respective group based on the median NLR among the patients within that group. These analyses were also repeated using group-specific optimal cutoffs calculated using an outcome-oriented approach based on the log-rank test statistic^27^.

#### Classification performance

We further assessed the ability of baseline NLR to predict overall, five year and ten year mortality by calculating the maximum sensitivity (S_1_; proportion of patients with high NLR accurately classified as deceased at the end of each time period) and specificity (S_2_; proportion of patients with low NLR accurately classified as alive at the end of each time period) via receiver operating characteristic curve analysis within the complete patient cohort. A combination of overall AUC and the sum of S_1_ and S_2_ was used to provide insights into not only the overall prognostic potential, but also the true and false positive rates that accompany specific thresholds for “high” versus “low” risk NLR. This analysis was repeated within the previously described patient subgroups (for example all black patients, or all male patients), and also for patient subgroups characterized by all possible combinations of 2 or 3 of our primary variable strata (for example all black male patients, or all black male patients over the age of 60). All subgroups containing 20 or more patients were included in these analyses. Predictive accuracy (as measured by S_1_, S_2_ and their sum (S_1_ + S_2_)) was compared across all groups to further validate for which patients the NLR may have the greatest prognostic potential. Groups in which S_1_ + S_2_ was at least 10% higher than in the cohort as a whole were highlighted. DeLong’s test for difference between ROC curves was used to validate the significant difference in AUC between patient subgroups, and demonstrate the increasing statistical significance of this difference as the patient subgroup is refined to incorporate more “high risk” demographic and/or clinical characteristics.

All statistical tests were two-sided, and statistical significance was defined as p < 0.05. All statistical analyses were performed using SAS 9.4 (SAS Institute Inc., Cary, NC) and R version 3.3.2 (R core development team, Vienna, Austria).

## Results

### Summary statistics

The 5,363 patients in the analytic sample had a mean age of 64 and were 50% male. Over 90% of the patients were non-Hispanic white. The average follow-up time for all patients was 3.7 years. Table 1 summarizes patient and tumor characteristics by primary cancer site.

**Table 1:** Data overview.

	Breast (N = 979)		Colorectal (N = 1024)		Esophageal (N = 594)		Liver (N = 281)		Melanoma (N = 349)		Ovarian (N = 245)		Pancreatic (N = 1276)		Prostate (N = 615)	
	#	%	#	%	#	%	#	%	#	%	#	%	#	%	#	%
**Age**																
Mean	58.9		63.8		66.5		66.1		61.4		64.7		66		65	
SD	13.6		13.5		10.4		11.8		16.4		11.8		11.3		8.6	
**Sex**																
Male	2	0.2%	523	51.1%	484	81.5%	210	74.7%	198	56.7%	0	0.0%	672	52.7%	615	100.0%
Female	977	99.8%	501	48.9%	110	18.5%	71	25.3%	151	43.3%	245	100.0%	604	47.3%	0	0.0%
**Race**																
White	856	87.4%	921	89.9%	572	96.3%	248	88.3%	343	98.3%	231	94.3%	1147	89.9%	532	86.5%
Black	75	7.7%	53	5.2%	10	1.7%	17	6.0%	3	0.9%	10	4.1%	76	6.0%	50	8.1%
Other	48	4.9%	50	4.9%	12	2.0%	16	5.7%	3	0.9%	4	1.6%	53	4.2%	33	5.4%
**Stage** (**TNM**)																
1	230	23.5%	145	14.2%	63	10.6%	20	7.1%	85	24.4%	11	4.5%	56	4.4%	2	0.3%
2	264	27.0%	169	16.5%	43	7.2%	7	2.5%	92	26.4%	5	2.0%	228	17.9%	61	9.9%
3	109	11.1%	191	18.7%	28	4.7%	7	2.5%	49	14.0%	86	35.1%	13	1.0%	13	2.1%
4	72	7.4%	153	14.9%	35	5.9%	6	2.1%	60	17.2%	67	27.3%	220	17.2%	10	1.6%
Unknown/Other	207	21.1%	272	26.6%	389	65.5%	229	81.5%	61	17.5%	63	25.7%	683	53.5%	507	82.4%
Missing	97	9.9%	94	9.2%	36	6.1%	12	4.3%	2	0.6%	13	5.3%	76	6.0%	22	3.6%
**Histology**																
Well differentiated	107	10.9%	68	6.6%	39	6.6%	66	23.5%	0	0.0%	6	2.4%	128	10.0%	20	3.3%
Moderately differentiated	359	36.7%	666	65.0%	197	33.2%	62	22.1%	0	0.0%	18	7.3%	207	16.2%	373	60.7%
Poorly differentiated	370	37.8%	131	12.8%	238	40.1%	37	13.2%	0	0.0%	102	41.6%	142	11.1%	19	3.1%
Undifferentiated	36	3.7%	14	1.4%	2	0.3%	2	0.7%	2	0.6%	67	27.3%	11	0.9%	198	32.2%
Not determined / Other	107	10.9%	144	14.1%	118	19.9%	114	40.6%	0	0.0%	51	20.8%	787	61.7%	2	0.3%
Missing	0	0.0%	1	0.1%	0	0.0%	0	0.0%	347	99.4%	1	0.4%	1	0.1%	3	0.5%
**Treatment type**^**a**^																
Surgery	847	86.5%	764	74.6%	235	39.6%	91	32.4%	283	81.1%	200	81.6%	412	32.3%	143	23.3%
Chemotherapy	577	58.9%	677	66.1%	459	77.3%	123	43.8%	57	16.3%	191	78.0%	953	74.7%	6	1.0%
Radiation Therapy	21	2.1%	330	32.2%	362	60.9%	51	18.1%	42	12.0%	0	0.0%	393	30.8%	400	65.0%
Immunotherapy	132	13.5%	152	14.8%	12	2.0%	5	1.8%	53	15.2%	1	0.4%	75	5.9%	0	0.0%
Hormone Therapy	511	52.2%	8	0.8%	4	0.7%	4	1.4%	4	1.1%	0	0.0%	28	2.2%	60	9.8%
Other	8	0.8%	6	0.6%	2	0.3%	4	1.4%	11	3.2%	0	0.0%	33	2.6%	0	0.0%
None	11	1.1%	18	1.8%	25	4.2%	41	14.6%	10	2.9%	4	1.6%	135	10.6%	32	5.2%

Summary of the demographic and clinical characteristics of the 5,363 patients included in the Moffitt Cancer Center cohort.

^a^Note that it is not uncommon for patients to receive multiple treatments, and thus the proportions of treatment type within each cancer may exceed 100%.

### Variation in NLR

**Figure 1:**
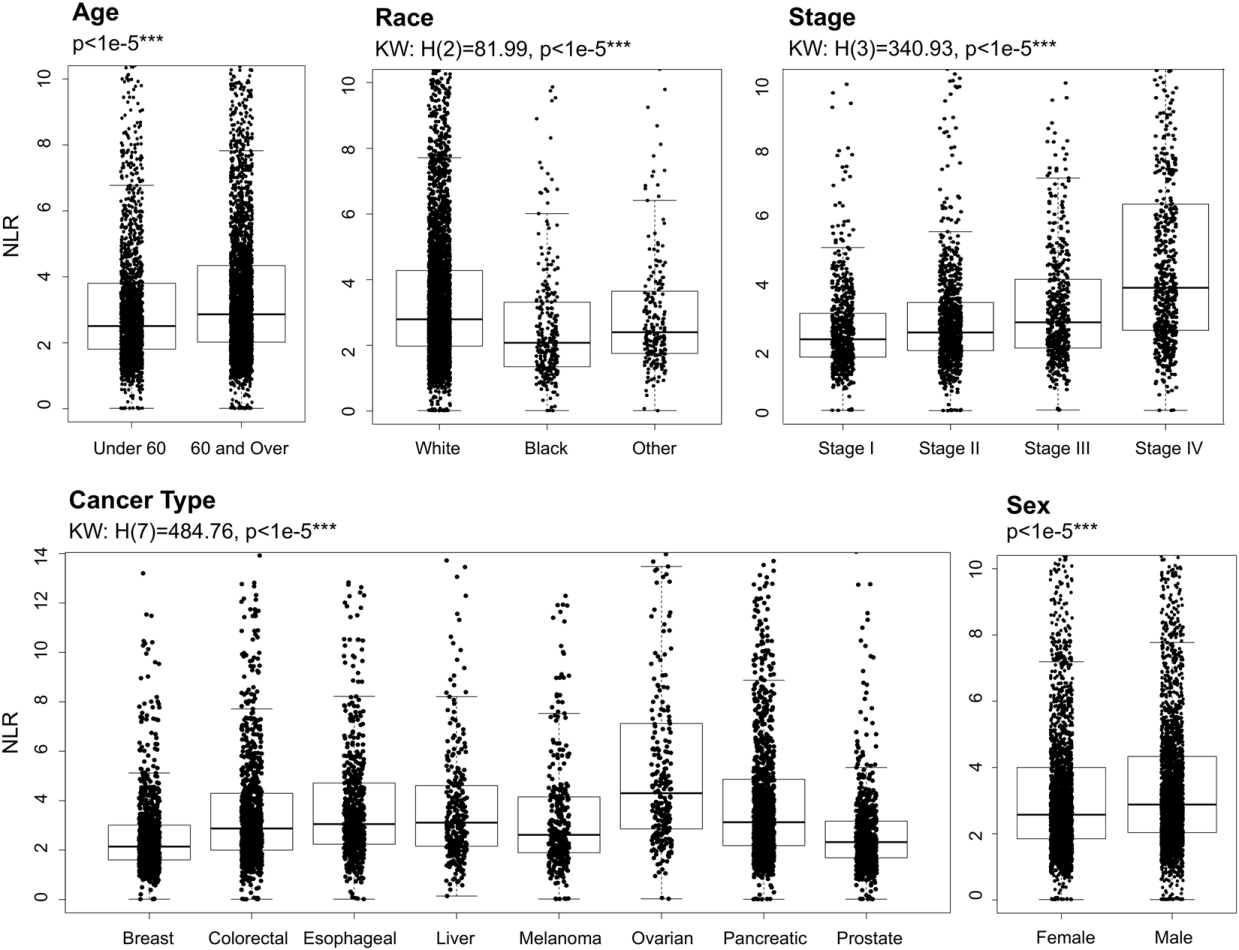
Variation in pre-treatment NLR. Box plots demonstrating baseline NLR in patients according to age, race, disease stage, cancer type and sex. Each point on the scatter plot represents an individual patient within the subgroup specified. The overlaid box plot presents the median and interquartile range of NLR for those patients.

Differences in baseline NLR among patient subgroups stratified by age, race, disease stage, cancer type and sex are summarized in Fig. 1. Patients aged 60 or over demonstrated a significantly higher baseline NLR than patients under 60 (2.86 versus 2.51, p < 1e-5). Male patients had significantly higher baseline NLR than female patients (2.88 and 2.57 respectively, p < 1e-5). Black patients exhibited lower NLR than white patients (2.08 as compared to 2.80, p < 1e-5), as did patients of other race (2.40 as compared to 2.80, p = 0.001). Stage IV patients demonstrated a significantly higher median baseline NLR than other stages of disease (3.75 as compared to 2.70 (stage III), 2.39 (stage II) and 2.18 (stage I), p < 1e-5 in all pairwise comparisons). Breast cancer and prostate cancer patients demonstrated the lowest pretreatment median NLR (2.14 and 2.32, respectively), and baseline NLR in ovarian cancer patients was particularly high at 4.30. Pairwise comparisons between each of these three respective sites and colorectal cancer, esophageal cancer, liver cancer, melanoma and pancreatic cancer were all statistically significant with p < 1e-5.

Comparable analyses were conducted for the absolute lymphocyte count (ALC) and absolute neutrophil count (ANC) components of the NLR; details can be found in Online Resource 1: S1a. Males and patients over 60 tended to have decreased peripheral blood ALC as compared to females and patients under 60, respectively (p < 1e-5 in both comparisons). White patients demonstrated significantly higher ANC than black patients (p < 1e-5). Stage IV patients exhibited both significantly higher ANC and significantly lower ALC than all other stages (p < 1e-5 in all comparisons), which together contributed to their significantly higher baseline NLR. Patients with breast and prostate cancer had both low ANC and high ALC, explaining their rank as the lowest NLR among the examined cancer sites. In patients with ovarian cancer, low ALC and particularly high ANC similarly explained the observed high NLR. Patients with liver cancer, however, had both the lowest ANC and the lowest ALC.

### Kaplan Meier analysis

**Figure 2:**
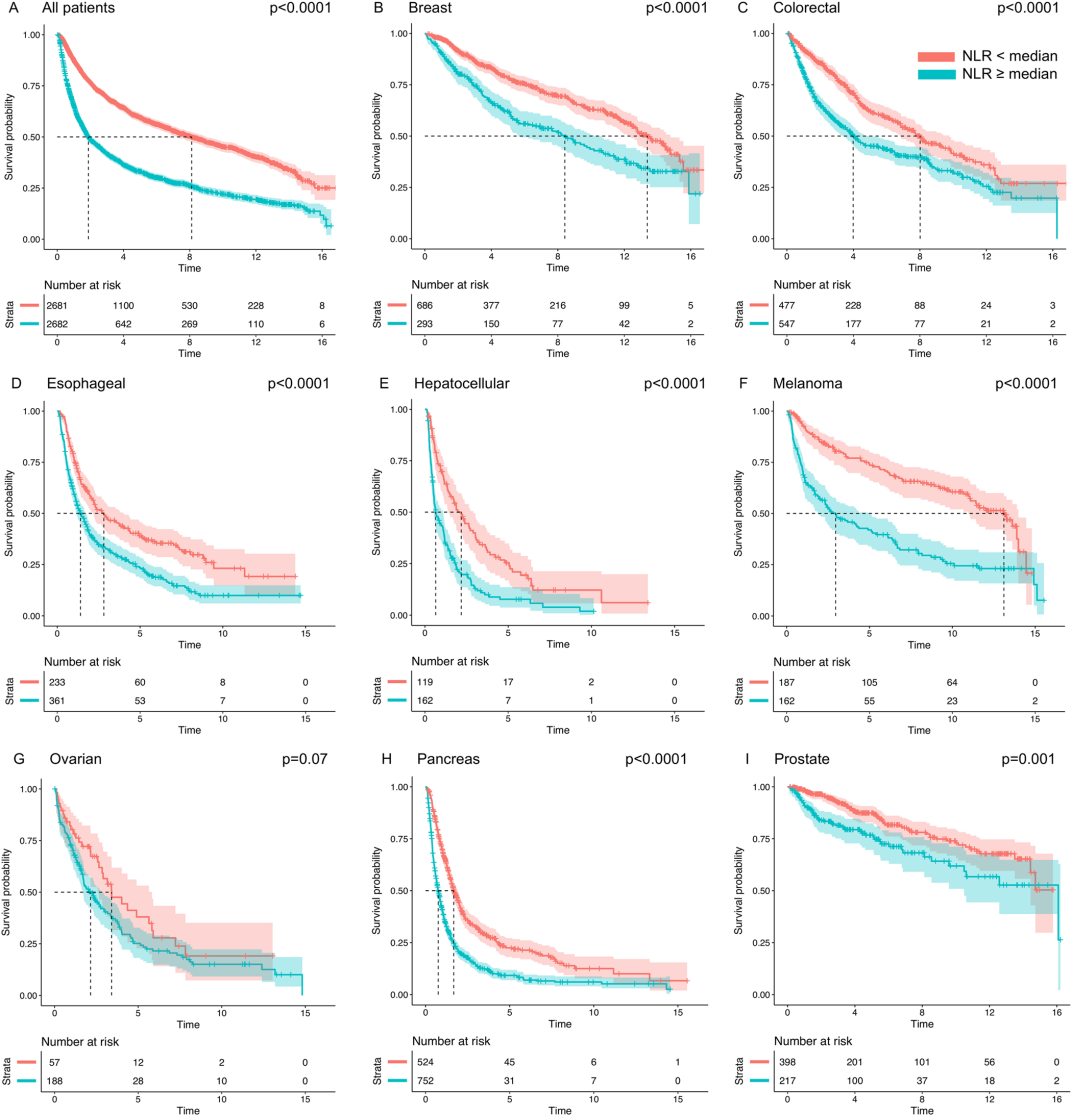
Survival Analysis. Kaplan Meier survival curves demonstrating survival probability with increasing time post diagnosis in patients with above-median NLR (blue) as compared to below-median NLR (red) in the cohort as a whole (**A**) and for each studied cancer type (**B–I**). The shaded areas reflect 95% confidence intervals. The hatched lines show median survival time for patients above or below the median NLR. Group-specific medians for determining high NLR cutoff are calculated within each cancer subtype.

Figure 2A presents results from log-rank tests and Kaplan Meier curves demonstrating the highly significant difference in overall survival between those patients with baseline NLR below the median of the whole cohort (NLR_med_ = 2.74) and those with baseline NLR equal to or above the median of the whole cohort. Patients were then divided into groups according to demographic and clinical characteristics (age, race, sex, disease stage, primary site, therapy type), and poorer overall survival in patients with above-median NLR was observed universally across cancer types (Fig. 2B–I) as well as across other subgroups (Online Resource 1: S1b and S1c). The magnitude of separation of the survival curves varied significantly. The median survival difference between above- and below-median NLR ranged from approximately 1 year in patients with pancreatic cancer to 10 years in patients with melanoma. We observed similar results for disease-specific survival (Online Resource 1: S2b and S2c).

### Cox proportional hazards analysis

Results from univariate analysis and corresponding multivariable Cox regression models assessing the association between NLR and overall survival are provided in Table 2. The cut-off for high NLR in this analysis is the overall cohort median NLR_med_. Log-rank tests for trend in ordinal variables (disease stage and differentiation status) were highly significant (p < 0.0001). With the exception of race, all variables demonstrated a significant association with survival in univariate analysis. In multivariable analysis, the adjusted HR for OS and high NLR was 1.58 [1.46–1.70]. Associations with stage, age, sex, disease type and differentiation status violated the proportional hazards assumption, and thus stratified multivariable regression was conducted for these variables. This stratification resulted in only minimal changes to the overall HR, which remained between 1.55 and 1.60 in all models, suggesting the proportional hazards violation does not significantly influence the magnitude of association between NLR and survival.

**Table 2:** Cox proportional hazards analysis: whole cohort.

	Overall Survival	
	Univariate	Multivariate^a^
	HR [95% CI]	HR [95% CI]
**Age**		
Under 60 (ref)	1	1
60 or Over	1.63 [1.50–1.76]	1.42 [1.31–1.55]
**Sex**		
Female [ref]	1	1
Male	1.20 [1.11–1.29]	1.01 [0.93–1.10]
**Race**		
White [ref]	1	
Black	0.95 [0.80–1.12]	
Other	0.95 [0.78–1.17]	
**Cancer Type**		
Breast [ref]	1	1
Colorectal	1.83 [1.60–2.11]	1.28 [1.08–1.52]
Esophageal	3.87 [3.35–4.48]	1.91 [1.58–2.3]
Hepatocellular	6.22 [5.23–7.38]	3.02 [2.45–3.73]
Melanoma	1.66 [1.39–1.99]	1.4 [1.12–1.75]
Ovarian	3.50 [2.90–4.23]	1.37 [1.09–1.71]
Pancreatic	6.35 [5.59–7.22]	3.19 [2.68–3.79]
Prostate	0.63 [0.52–0.78]	0.23 [0.18–0.30]
**Stage (TNM)**		
1 [ref]	1	1
2	1.46 [1.22–1.75]	1.51 [1.26–1.81]
3	2.14 [1.77–2.58]	2.29 [1.87–2.80]
4	6.39 [5.39–7.57]	3.57 [2.95–4.32]
**Histology**		
Well differentiated [ref]	1	1
Moderately differentiated	0.89 [0.76–1.04]	1.47 [1.25–1.72]
Poorly differentiated	1.44 [1.23–1.68]	2.14 [1.81–2.51]
Undifferentiated	1.65 [1.28–2.15]	2.38 [1.80–3.13]
**Treatment type**		
Surgery	0.34 [0.32–0.37]	0.39 [0.35–0.43]
Chemotherapy	1.82 [1.69–1.96]	0.93 [0.90–1.03]
Radiation therapy	0.62 [0.57–0.67]	0.77 [0.70–0.84]
Immunotherapy	1.03 [0.90–1.18]	0.79 [0.69–0.92]
**NLR**		
<Median [ref]	1	1
>Median	2.21 [2.05–2.38]	**1.58 [1.46–1.70]**

Hazard ratios and 95% confidence intervals from univariate and multivariable Cox proportional hazards models for NLR greater than or equal to the whole cohort median.

^a^All covariates significant in univariate analysis are included in the multivariable model(s).

The multivariable analysis was repeated to assess the strength of independent association of high NLR and overall survival within each of our previously described subgroups of interest. Table 3 presents multivariate-adjusted HRs and 95% CIs for the association between NLR and OS in each patient subgroup (HR’s and 95% CI for covariates are not shown for these 81 analyses). A high NLR was associated with a 49% higher hazard of mortality (HR = 1.49, CI = 1.33–1.67) in females and a 60% higher hazard of mortality (HR = 1.60, CI = 1.44–1.78) in males. In black patients (HR = 2.07, CI = 1.43–3.00), stage IV patients (HR = 2.14, CI = 1.78–2.58) and patients receiving only chemotherapy (HR = 1.98, CI = 1.69–2.32), the association between NLR and OS was stronger than that in the cohort as a whole. This association with OS was similarly stronger in patients with liver (HR = 1.92, CI = 1.44–2.56), ovarian (HR = 1.68, CI = 1.21–2.33) and pancreatic cancer (HR = 1.74, CI = 1.53–1.98) as well as in melanoma patients (HR = 1.73, CI = 1.27–2.37). We further evaluated these associations for five year and ten year overall survival to more accurately capture relationships in groups with poorer prognoses (for example, esophageal, liver, ovarian and pancreatic cancer all have fewer than 20 patients still undergoing follow up after 10 years) as shown in Table 3. Here, black patients, stage IV patients, patients receiving only radiation therapy and melanoma patients exhibit hazard ratios in excess of 2, well above the point estimate for the population as a whole.

**Table 3:** Cox proportional hazards analysis: subgroups.

	Overall Survival (By Group)	5 Year Overall Survival (By Group)	10 Year Overall Survival (By Group)
	Multivariate	Multivariate	Multivariate
	HR [95% CI]	HR [95% CI]	HR [95% CI]
**Age**			
Under 60	1.50 [1.29–1.74]	1.55 [1.32–1.82]	1.51 [1.30–1.75]
60 or Over	1.57 [1.44–1.72]	1.66 [1.50–1.83]	1.61 [1.47–1.77]
**Sex**			
Female	1.49 [1.33–1.67]	1.61 [1.42–1.82]	1.60 [1.44–1.78]
Male	1.60 [1.44–1.78]	1.61 [1.44–1.81]	1.55 [1.38–1.74]
**Race**			
White	1.57 [1.45–1.70]	1.64 [1.51–1.80]	1.60 [1.48–1.74]
Black	2.07 [1.43–3.00]	2.08 [1.41–3.06]	2.06 [1.41–3.00]
Other	1.22 [0.76–1.95]	1.42 [0.86–2.33]	N/A
**Cancer Type**			
Breast	1.25 [0.99–1.56]	1.35 [1.01–1.79]	1.26 [0.98–1.60]
Colorectal	1.38 [1.14–1.66]	1.48 [1.20–1.83]	1.38 [1.14–1.67]
Esophageal	1.57 [1.29–1.92]	1.60 [1.30–1.97]	N/A
Hepatocellular	1.92 [1.44–2.56]	1.88 [1.41–2.53]	N/A
Melanoma	1.73 [1.27–2.37]	2.02 [1.37–2.97]	2.02 [1.44–2.82]
Ovarian	1.68 [1.21–2.33]	1.81 [1.28–2.57]	N/A
Pancreatic	1.74 [1.53–1.98]	1.77 [1.55–2.02]	N/A
Prostate	1.35 [0.93–1.97]	1.78 [1.09–2.88]	1.47 [0.99–2.20]
**Stage (TNM)**			
1	1.18 [0.88–1.60]	1.11 [0.75–1.64]	1.12 [0.81–1.55]
2	1.39 [1.11–1.74]	1.42 [1.08–1.87]	1.38 [1.09–1.75]
3	1.33 [1.00–1.75]	1.56 [1.14–2.14]	1.37 [1.03–1.81]
4	2.14 [1.78–2.58]	2.17 [1.80–2.62]	N/A
**Histology**			
Well differentiated	1.41 [1.05–1.89]	1.40 [1.00–1.96]	1.36 [1.00–1.85]
Moderately differentiated	1.44 [1.24–1.68]	1.53 [1.29–1.82]]	1.47 [1.26–1.72]
Poorly differentiated	1.47 [1.25–1.72]	1.47 [1.24–1.76]	1.46 [1.24–1.73]
Undifferentiated	1.27 [0.74–2.19]	1.25 [0.69–2.26]	1.29 [0.74–2.24]
**Treatment type**			
Surgery only	1.33 [1.10–1.60]	1.55 [1.23–1.96]	1.33 [1.09–1.63]
Chemotherapy only	1.98 [1.69–2.32]	N/A	N/A
Radiation therapy only	1.40 [0.85–2.32]	2.08 [1.08–4.00]	1.70 [0.98–2.94]
Immunotherapy only	2.60 [0.58–11.70]	N/A	N/A

Adjusted HRs and 95% CIs calculated within each patient subgroup, demonstrating the variation in strength of association between high NLR (>median) and survival outcomes (overall survival, five year overall survival, ten year overall survival) between patients with different demographic and clinical characteristics.

Note that adjusted HRs and 95% Cis are presented for 81 independent analyses (3 survival measures within 27 defined patient subgroups); covariates within each model are not shown.

N/A entries signify groups in which less than 20 patients were available for analysis.

Analyses were also conducted for disease-specific survival as the endpoint using the group-specific median cutoff for high NLR; findings were similar to those for OS (Online Resource 1: S1d). As an additional test, these medians were listed alongside optimal cutoffs for the continuous NLR variable in each group identified by the maximal log-rank test statistic method, and multivariable proportional hazards analysis was repeated for both overall and disease-specific survival using these optimal cutoffs (Online Resource 1: S1e). Almost universally across patient subgroups, hazard ratios for overall and disease-specific survival increased when the NLR cut-off was calculated using this minimum p-value method (Online Resource 1: S1e).

### Classification performance

**Figure 3:**
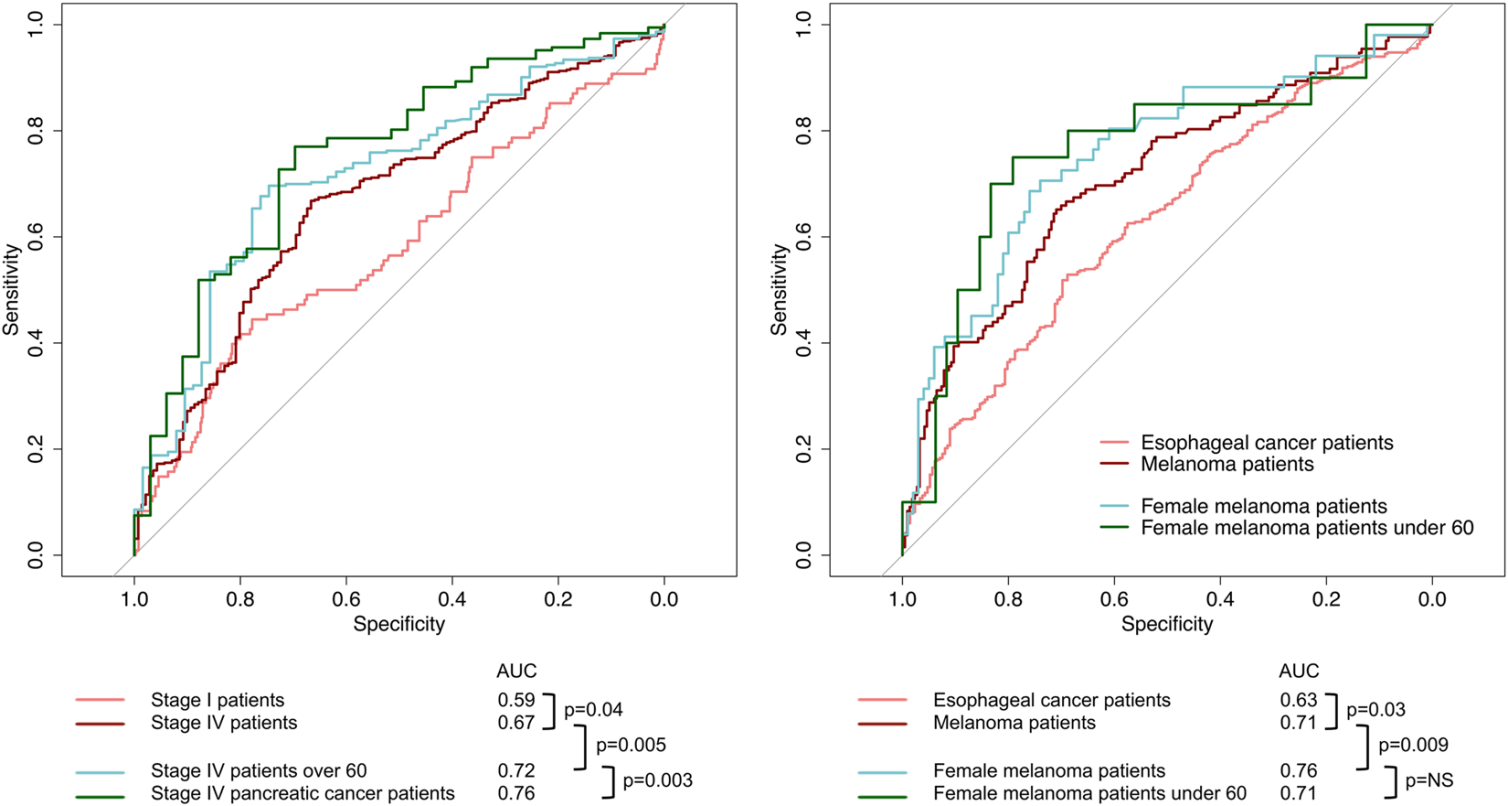
Prognostic potential of NLR. ROC curves demonstrating the diagnostic potential (5-year overall survival) of NLR as a binary classifier. Each colored curve represents a different patient subgroup. The AUC is increased in patients with stage IV disease (**A**) and melanoma (**B**) as compared to those with lower stage disease and/or other cancer types. In the example, the accuracy of NLR as a prognostic marker is increased significantly in patients exhibiting multiple “high risk” characteristics.

For the patient cohort as a whole, NLR as a predictor of overall patient survival demonstrated a maximum sensitivity S_1_ and specificity S_2_ of 0.75 and 0.51 (S_1_ + S_2_ = 1.26), respectively, for an optimal cut-off of NLR = 3.22. Results were similar for 5 year (S_1_ + S_2_ = 1.28, cut-off = 3.27) and 10 year (S_1_ + S_2_ = 1.26, cut-off = 3.22) overall survival. Considering all possible combinations of 1, 2 or 3 strata of our primary variables age, race, sex, stage and cancer type resulted in a total of 451 patient subgroups for analysis. For overall survival, 32 of these patient subgroups demonstrated an increase in the prognostic potential of the NLR (as measured by S_1_ + S_2_) of more than 10% (S_1_ + S_2_ > 1.4) as compared to the cohort as a whole (S_1_ + S_2_ = 1.26–1.28). For 5 year and 10 year overall survival, 60 and 57 patient subgroups met this criterion, respectively. Almost exclusively, groups with S_1_ + S_2_ > 1.4 (149 total) featured patients of non-white race, females, stage III or IV patients, and/or melanoma or pancreatic cancer patients, with only 12 exceptions (8%). Figure 3 demonstrates the difference in prognostic accuracy of the NLR between strata of individual variables (stage I vs stage IV patients, esophageal vs melanoma patients) and shows the increased sensitivity and specificity of NLR when multiple “high risk” characteristics are present. DeLong’s test for difference in ROC curves demonstrated sequentially increasing AUC and sequentially decreasing p-values (Fig. 3A,B) as the patient subgroup is refined, with the exception of female melanoma patients over 60 where the difference in AUC became non-significant due to diminishing sample size. Interactions between these respective patient characteristics (non-white race, females, stage III or IV patients, and/or melanoma or pancreatic cancer patients) were also evaluated within the Cox proportional hazards model. Interaction term coefficients were significant for four combinations of characteristics: female sex and age > 60 (*β* = 0.17, *p* = 0.04); female sex and black race (*β* = 0.18, *p* = 0.04); age > 60 and melanoma diagnosis (*β* = 0.52, *p* = 0.01); and stage IV disease and melanoma diagnosis (*β* = 0.77, *p* = 0.02).

## Discussion

While a large body of literature exists on the prognostic potential of the NLR, the translation of this promising marker into the clinical setting remains highly challenging as the strength of association between NLR and overall survival varies dramatically between published studies^6^. Here, we used a large retrospective cohort of patients treated at Moffitt Cancer Center to take a more detailed look at the association between NLR and survival outcomes in demographically and clinically homogeneous patient subgroups, compare the findings to those presented in the existing literature, and identify the patients for whom the NLR may have maximum prognostic power.

Results suggest that average values of the NLR vary significantly between subgroups of the population, and the magnitude of association between high NLR and survival outcomes is greater for certain patients than for the cohort as a whole. Baseline NLR tends to be significantly higher in white patients, male patients and over 60 s, and particularly in patients with stage IV disease and patients with ovarian and pancreatic cancer. Analysis of the ALC and ANC components suggested that the immunological mechanisms driving these differences in baseline NLR are complex, with substantial variation between patient subgroups in the contribution of each cell type to the overall ratio. Some of these differences have already been addressed in the literature, for example we observed a significantly lower absolute neutrophil count in black patients than white patients, consistent with existing observations of benign ethnic neutropenia^22^, and the decreasing capacity for a strong adaptive immune response with age has been well documented^23^. However, further analysis of the biological mechanisms underlying these differences should be a priority for further study, particularly between cancer type subgroups where the differences are particularly striking.

High baseline values of the NLR do not necessarily translate into the strongest associations with survival outcomes. In unadjusted Kaplan Meier analysis, a survival difference of over 10 years was observed comparing melanoma patients with baseline NLR above the group-specific median to those with baseline NLR below the group-specific median. For pancreatic cancer patients, this survival difference was only around one year. These results provided an early indication that the baseline NLR may have greater clinical value for certain individuals based on their clinical and/or demographic characteristics. Black patients, patients receiving only radiation therapy, melanoma patients, and stage IV patients all exhibited substantially higher adjusted hazard ratios for high NLR and overall and disease-specific survival than the population as a whole; these may be promising patient populations for early attempts at prospective validation of the prognostic value of the NLR. Although associations with survival outcomes become even stronger using ROC-calculated “optimal” cutoffs for high-risk NLR as compared to the group-specific median, as previously mentioned, the clinical utility of such thresholds is uncertain.

We evaluated whether these increased hazard ratios could translate into greater prognostic power in certain groups of patients, with an increased ability to accurately predict patient mortality. Within patient subgroups defined by disease stage, race, and certain cancer types including melanoma—the same subgroups that tended to exhibit stronger associations with survival outcomes—we observed consistently higher AUC. We noted an even more significant increase in the prognostic power (as measured by AUC) of the NLR among patients who had two or more of the clinical or demographic characteristics found to have the strongest associations with outcome.

The assumption that the NLR has equal prognostic value for all patients regardless of demographic factors or clinical characteristics of the disease is highly likely to be incorrect. Future work should emphasize the identification and validation of clinically meaningful thresholds for risk stratification within “high-risk” patient subgroups, facilitating prospective evaluation of the prognostic power of the NLR within these groups to determine whether clinical implementation of the NLR as a prognostic tool is a realistic and attainable goal. Larger within-institution studies of the association between high NLR and outcomes across patients with a range of clearly defined demographic and clinical characteristics could also reduce the number of potential sources of variation in effect size, which are inherent in the post-hoc comparison of many independent small-scale studies. Steps toward establishing universal thresholds for high NLR to improve comparability of study results could also include improved transparency in reporting, and presenting exploratory results using several cutoffs or sharing data sets (or more detailed summary statistics) could allow future meta-analyses to impose universal thresholds or treat the NLR as a continuous variable and help to strengthen the conclusions that can be drawn from existing data. The association between NLR and outcomes also varies between molecular subgroups^28,29^; while this was beyond the scope of the present study, an additional future direction may be further exploration into the relationships between NLR and molecular profiles that may influence treatment response and patient outcomes. Finally, extension of the analysis to a larger number of cancer types, and further stratification of the patient cohort by specific therapy combination and sequence could lead to additional insights also beyond the scope of the present work.

The routine collection of the complete blood count in clinical practice at minimal cost and inconvenience to the patient makes the NLR a highly promising marker for the systemic inflammatory status of the patient. If this potential can be harnessed to permit clinical risk stratification at diagnosis in even a small subpopulation of cancer patients, particularly when coupled with a deeper understanding of the underlying immune mechanisms governing the relative contributions of the ANC and ALC, there could be potential to guide patient-specific therapeutic interventions for improved survival outcomes.

## Supplementary information

Supplementary Material

## Data Availability

The datasets analyzed during the current study are not publicly available as they contain identifiable protected health information (PHI).
