# Supplementary material for: Exploring the prognostic value of the neutrophil-to-lymphocyte ratio in cancer
Source: Sci Rep. 2019 Dec 23;9:19673. doi: 10.1038/s41598-019-56218-z (PMC6928022; doi:10.1038/s41598-019-56218-z)
Supplement: Supplementary file 1 — Supplementary Material [file 41598_2019_56218_MOESM1_ESM.pdf]

Exploring the prognostic value of the neutrophil-to-lymphocyte ratio in cancer.

Rachel Howard Ph.D.*, Peter A. Kanetsky Ph.D., Kathleen M. Egan Ph.D.

Department of Cancer Epidemiology

H. Lee Moffitt Cancer Center and Research Institute, Tampa, FL

*Correspondence:

12902 USF Magnolia Drive, MRC2

Tampa, FL, 33612

rachel.howard@moffitt.org

(+1) 813-745-4651

Supplementary Material

S1a. Baseline lymphocyte and neutrophil counts

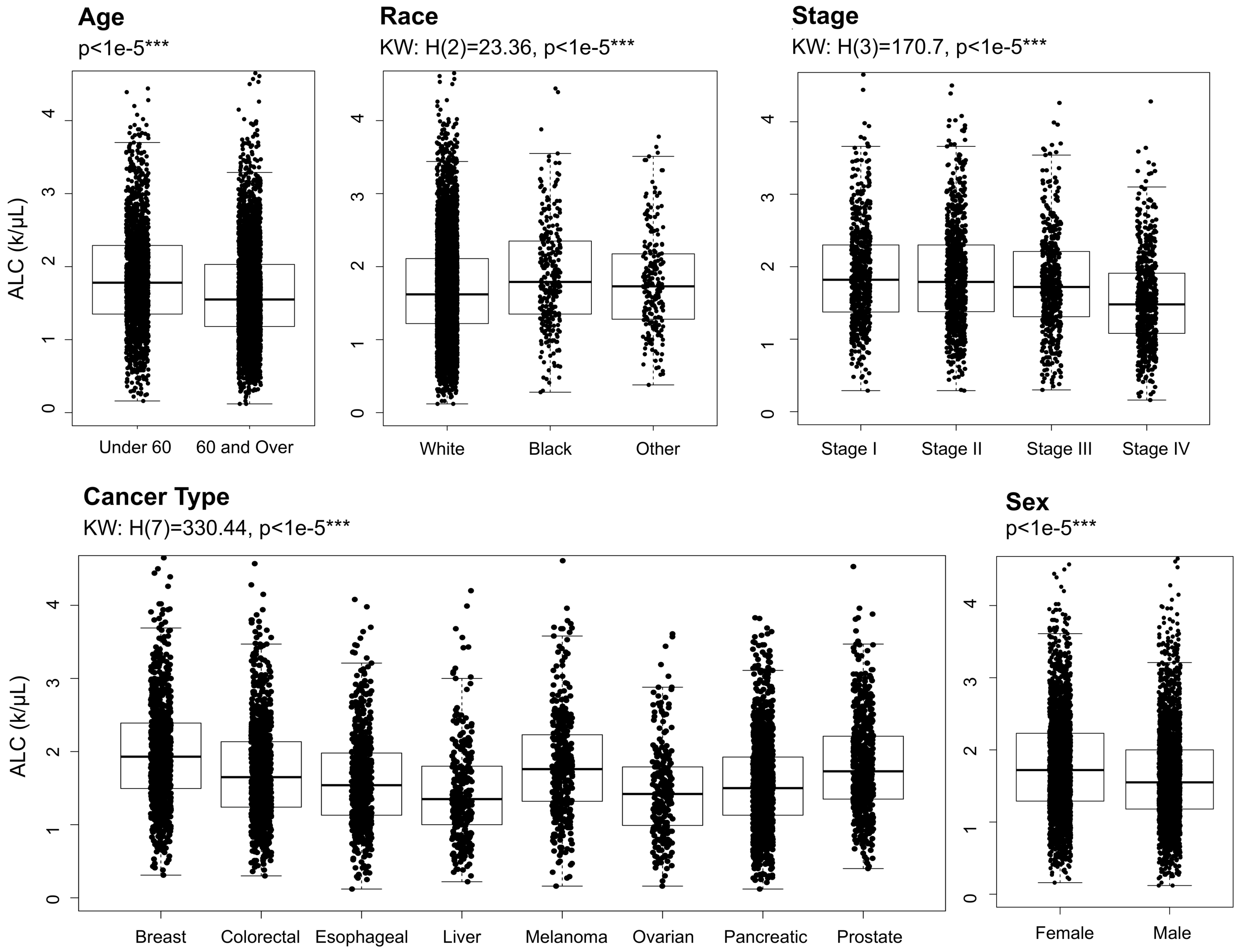

Figure S1. Box plots demonstrating the differences in baseline absolute lymphocyte count (ALC) between patient subgroups stratified by age, race, disease stage, cancer type and sex.

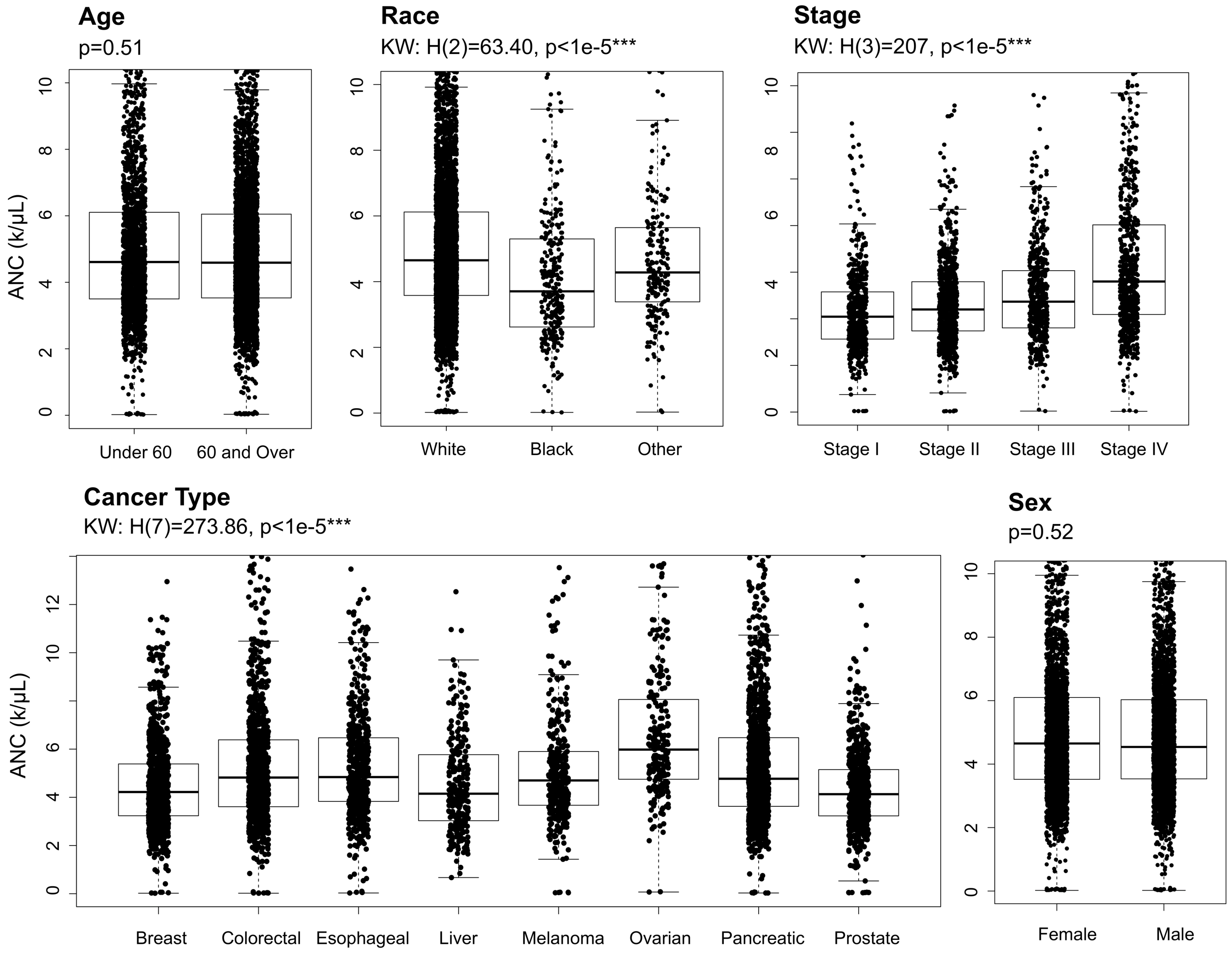

Figure S2. Box plots demonstrating the differences in baseline absolute neutrophil count (ANC) between patient subgroups stratified by age, race, disease stage, cancer type and sex.

S1b. Kaplan meier survival curves: Overall survival

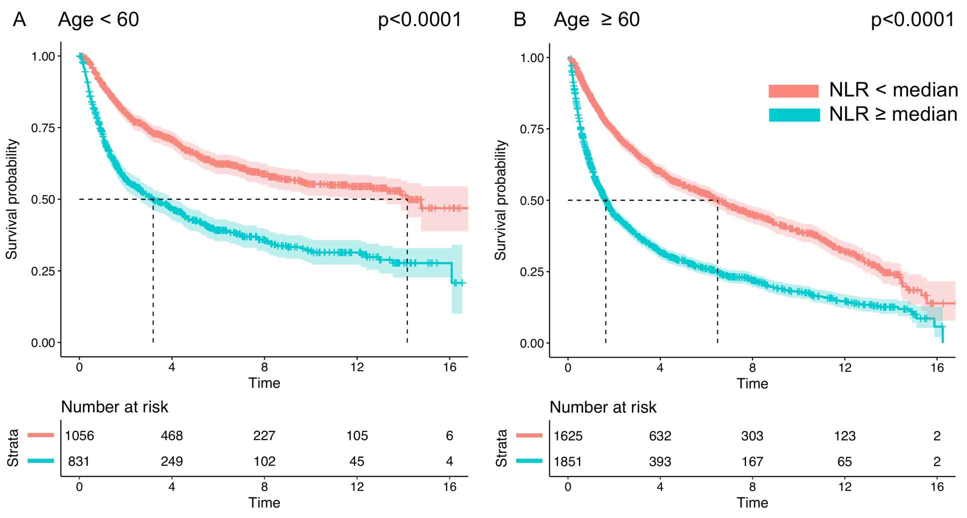

Figure S3. Kaplan meier survival curves demonstrating differences in overall survival outcomes between patients with above-median NLR (blue) as compared to below-median NLR (red) in patients under 60 (**A**) and patients 60 or over (**B**).

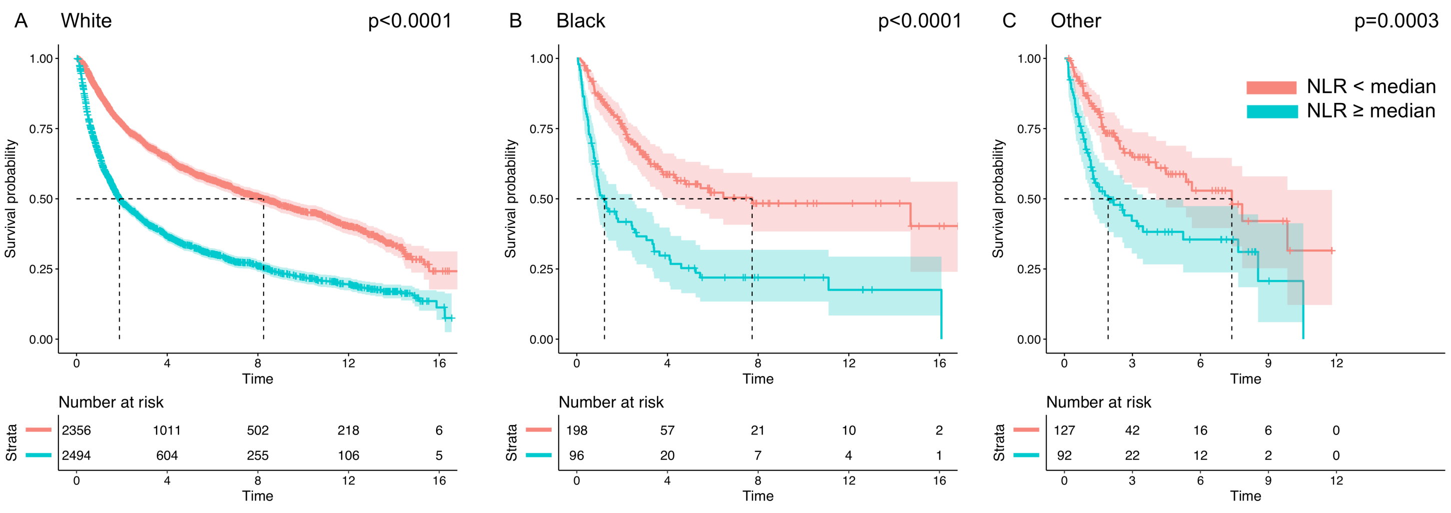

Figure S4. Kaplan meier survival curves demonstrating differences in overall survival outcomes between patients with above-median NLR (blue) as compared to below-median NLR (red) in white patients (**A**), black patients (**B**) and patients of other race (**C**).

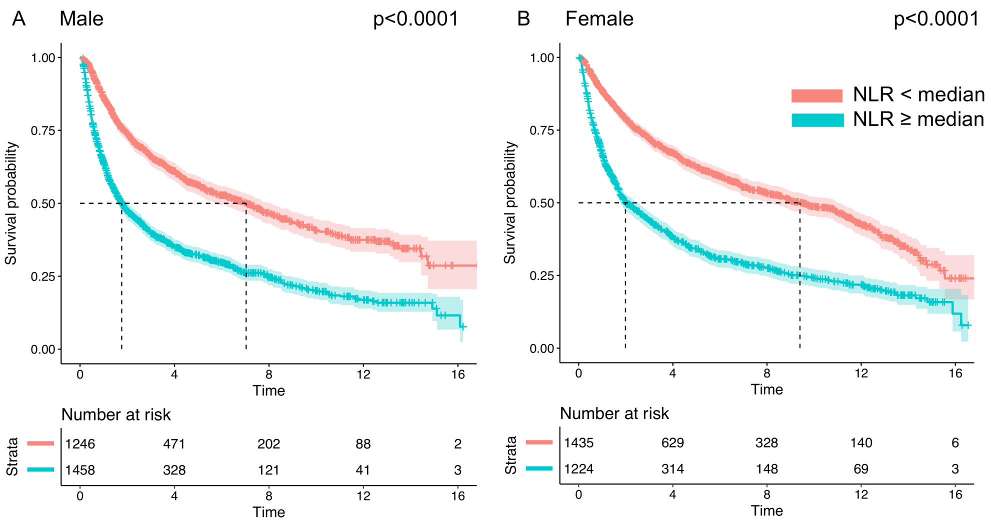

Figure S5. Kaplan meier survival curves demonstrating differences in overall survival outcomes between patients with above-median NLR (blue) as compared to below-median NLR (red) in male patients (**A**) and female patients (**B**).

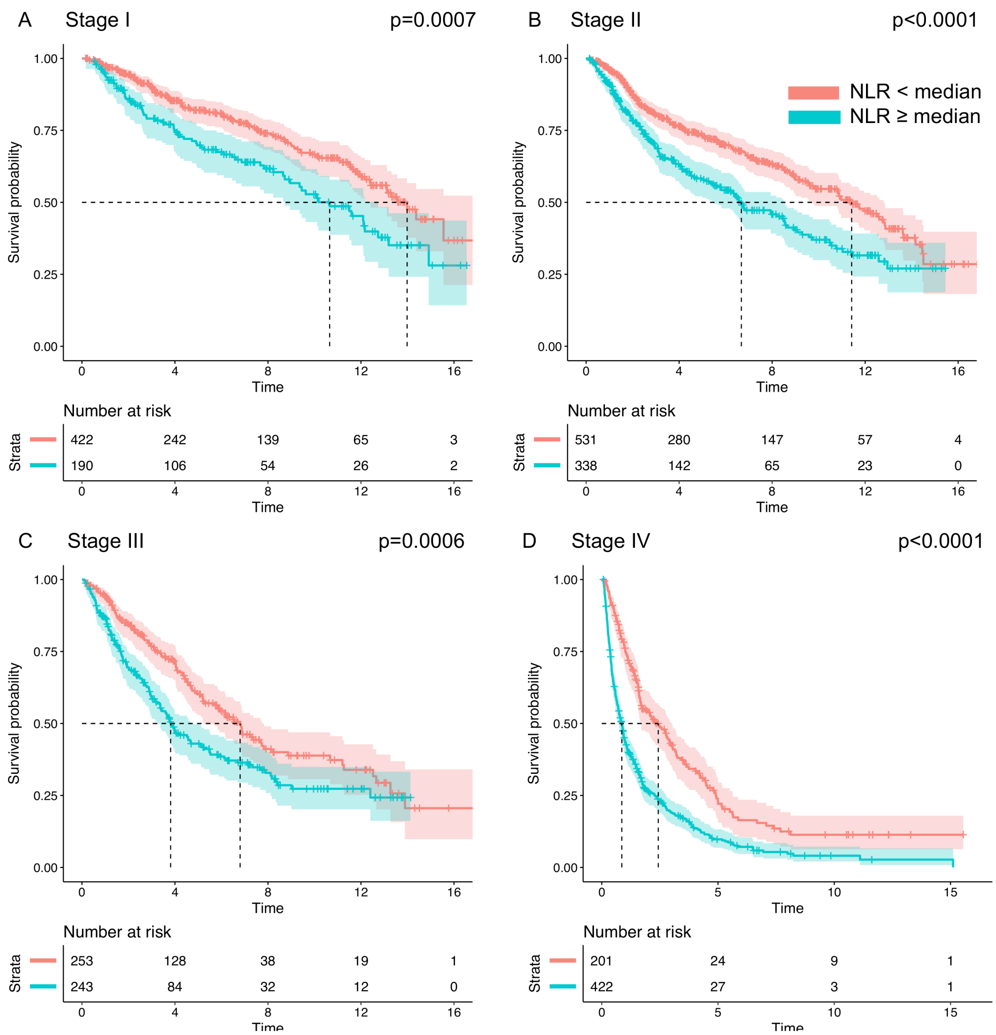

Figure S6. Kaplan meier survival curves demonstrating differences in overall survival outcomes between patients with above-median NLR (blue) as compared to below-median NLR (red) in stage I patients (**A**), stage II patients (**B**), stage III patients (**C**) and stage IV patients (**D**).

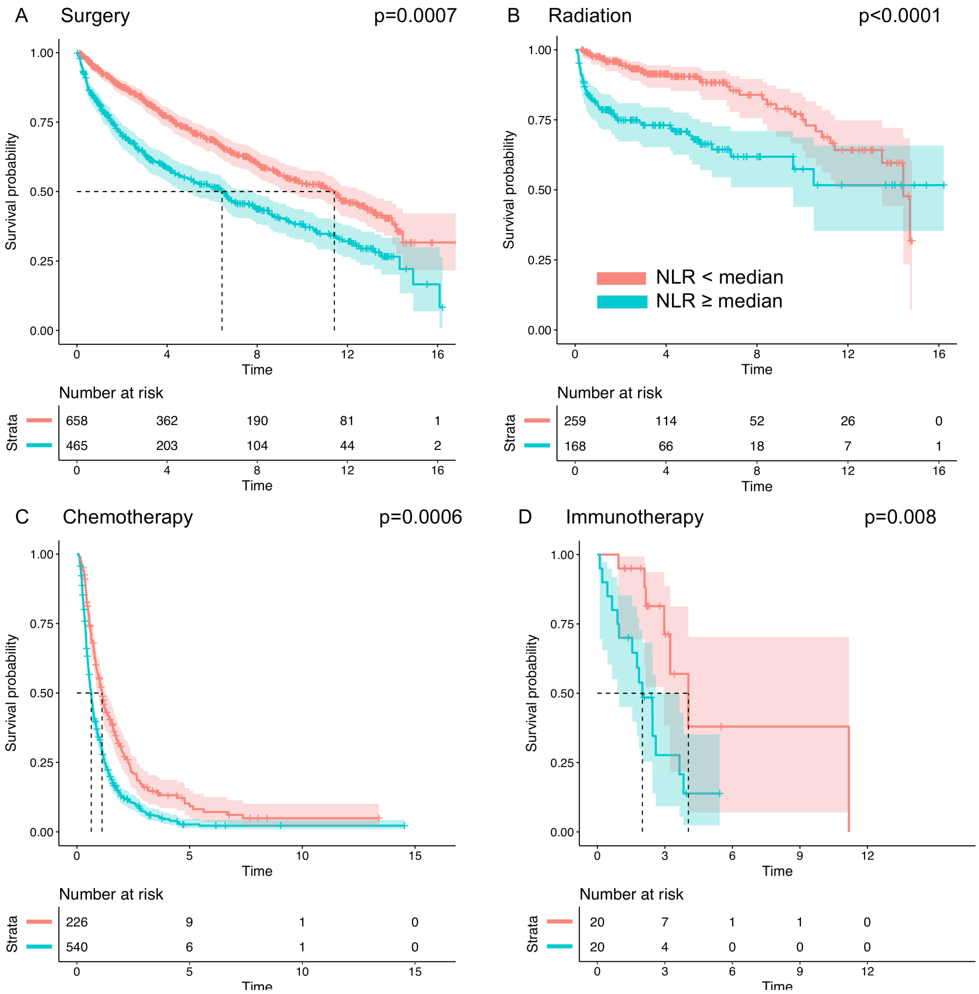

Figure S7. Kaplan meier survival curves demonstrating differences in overall survival outcomes between patients with above-median NLR (blue) as compared to below-median NLR (red) in patients receiving only surgery (**A**), patients receiving only radiation therapy (**B**), patients receiving only chemotherapy (**C**) and patients receiving only immunotherapy (**D**).

S1c. Kaplan meier survival curves: Disease-specific survival

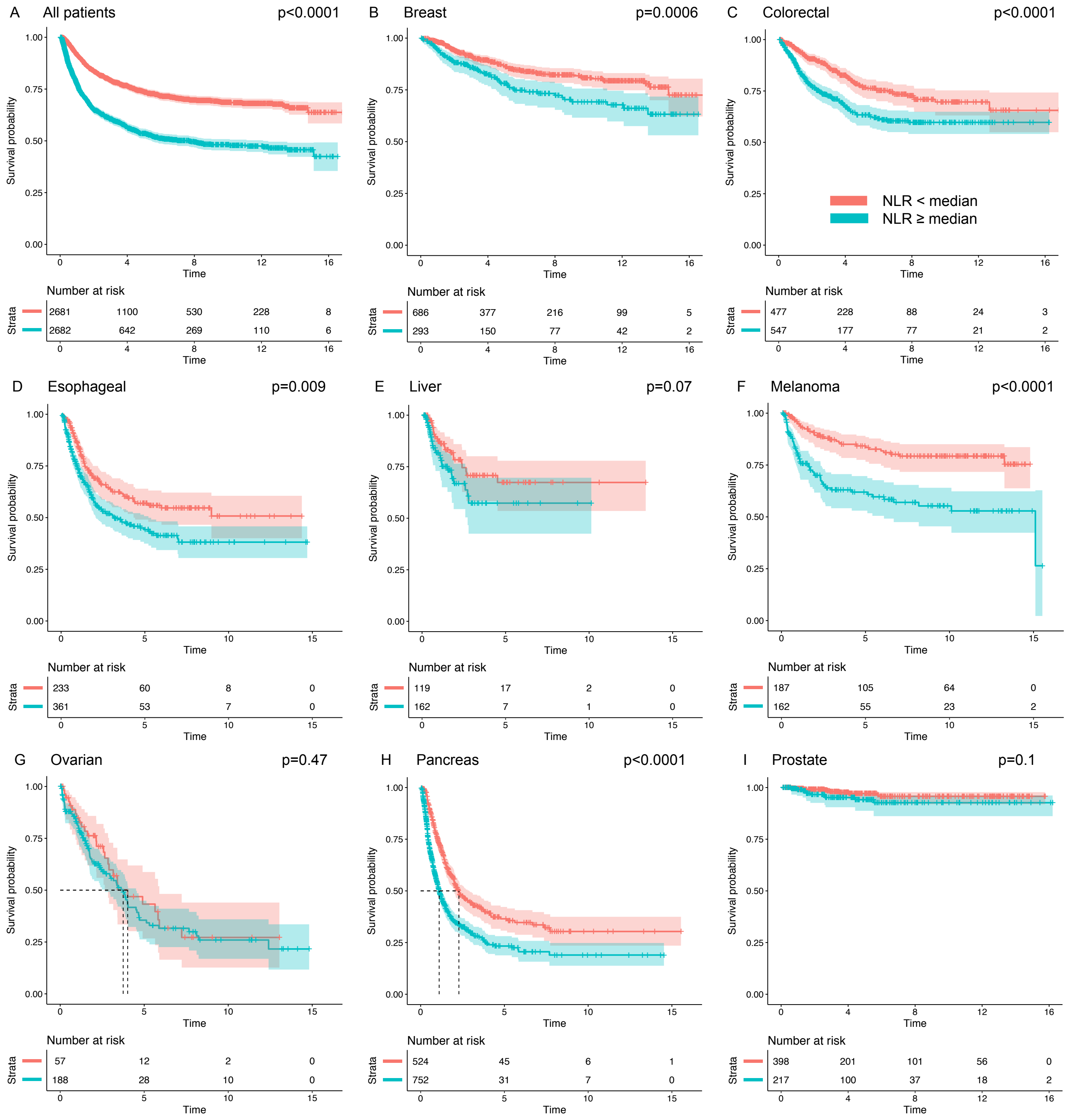

Figure S8. Kaplan meier survival curves demonstrating differences in disease-specific survival outcomes between patients with above-median NLR (blue) as compared to below-median NLR (red) in all patients (**A**) and subgroups characterized by shared cancer type (**B-I**).

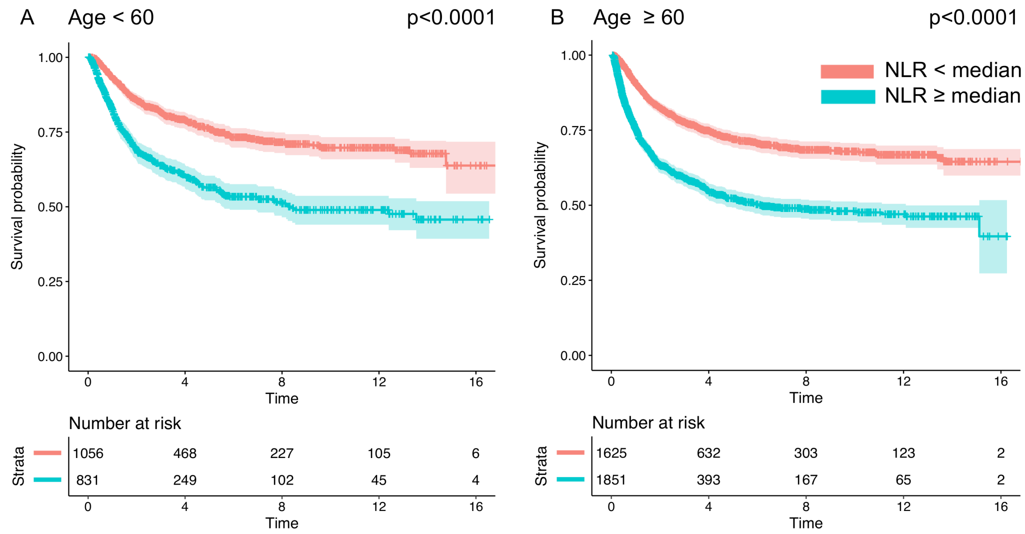

Figure S9. Kaplan meier survival curves demonstrating differences in disease-specific survival outcomes between patients with above-median NLR (blue) as compared to below-median NLR (red) in patients under 60 (**A**) and patients aged 60 or over (**B**).

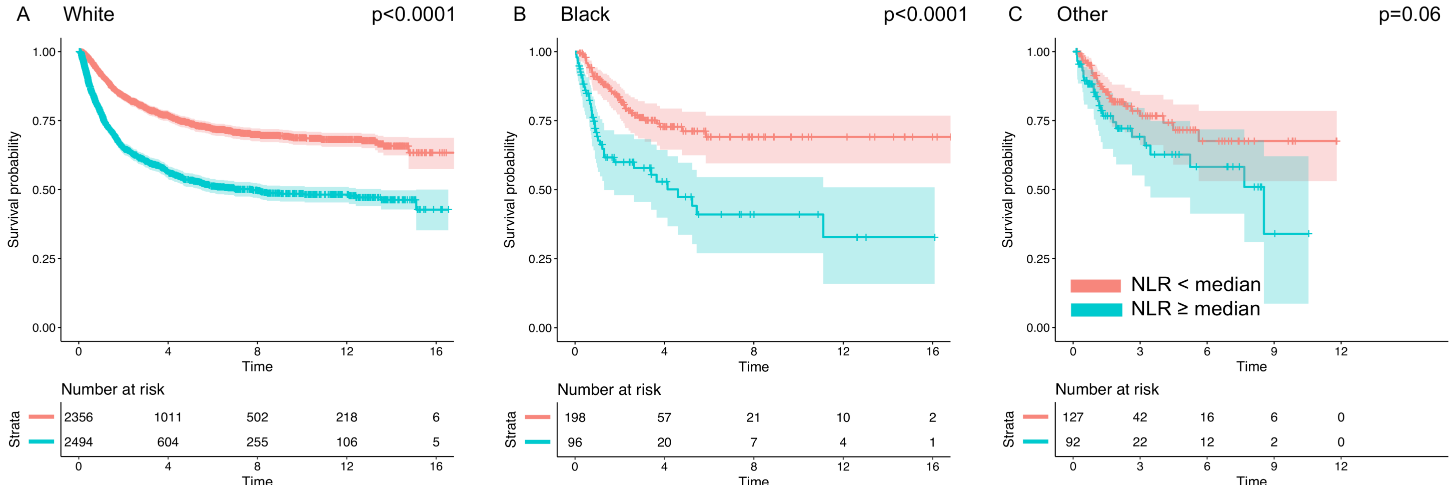

Figure S10. Kaplan meier survival curves demonstrating differences in disease-specific survival outcomes between patients with above-median NLR (blue) as compared to below-median NLR (red) in white patients (**A**), black patients (**B**), and patients of other race (**C**).

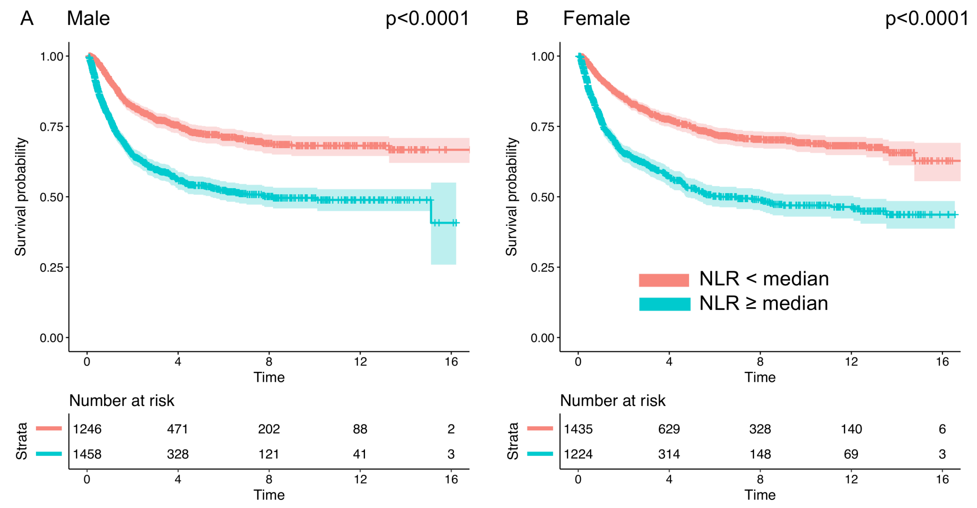

Figure S11 Kaplan meier survival curves demonstrating differences in disease-specific survival outcomes between patients with above-median NLR (blue) as compared to below-median NLR (red) in male patients (**A**) and female patients (**B**).

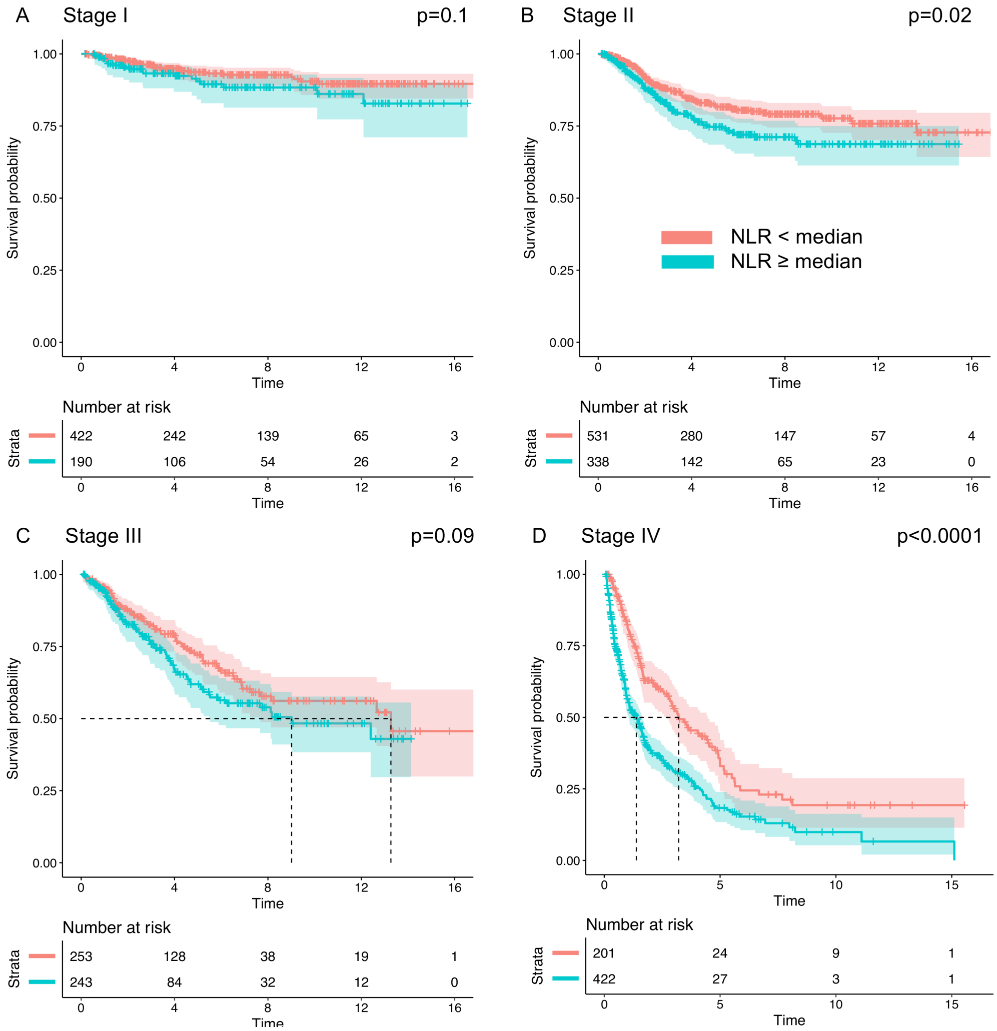

Figure S12. Kaplan meier survival curves demonstrating differences in disease-specific survival outcomes between patients with above-median NLR (blue) as compared to below-median NLR (red) in stage **I** patients (**A**), stage **II** patients (**B**), stage **III** patients (**C**), and stage IV patients (**D**).

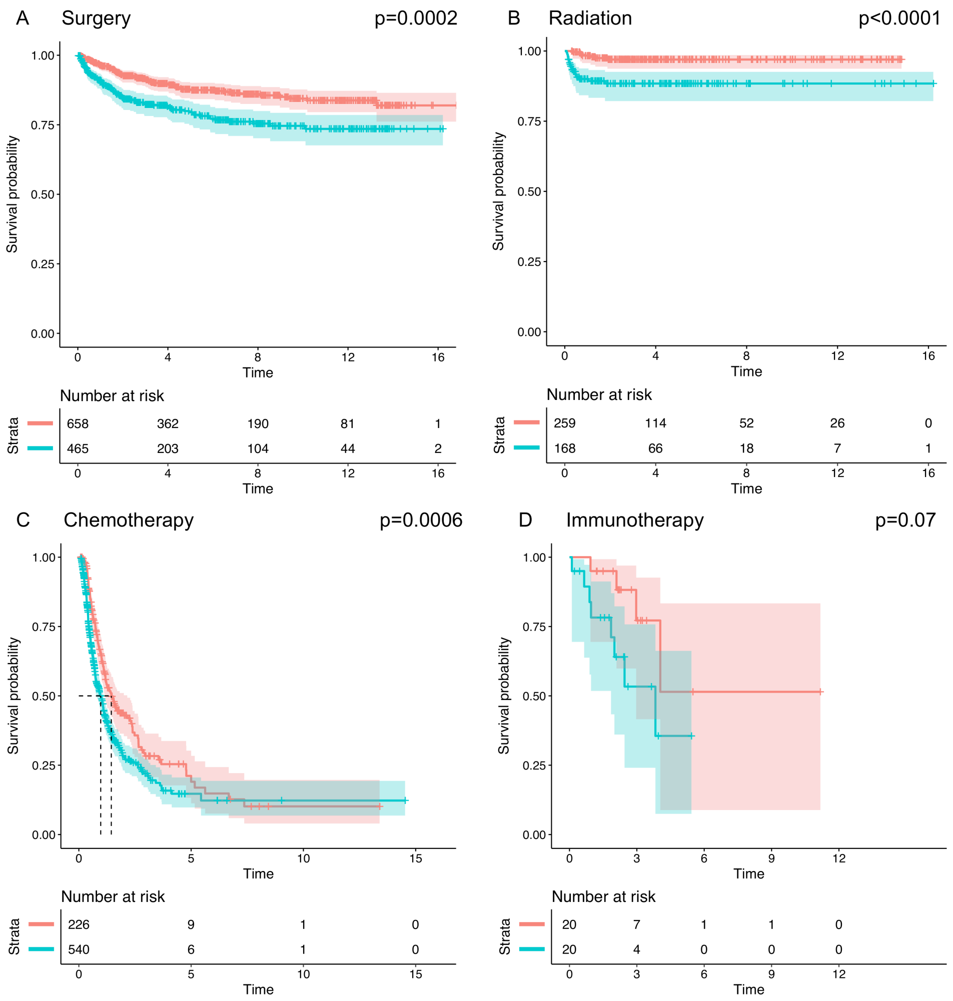

Figure S13. Kaplan meier survival curves demonstrating differences in disease-specific survival outcomes between patients with above-median NLR (blue) as compared to below-median NLR (red) in patients receiving only surgery (**A**), patients receiving only radiation therapy (**B**), patients receiving only chemotherapy (**C**), and patients receiving only immunotherapy (**D**).

S1d. Multivariable Cox proportional hazards analysis for high NLR and disease-specific survival

A

	Disease-Specific Survival	
	Univariate HR [95% CI]	Multivariate HR [95% CI]
**Age**		
Under 60 (ref)	1	1
60 or Over	1.26 [1.13-1.40]	1.18 [1.05-1.31]
**Sex**		
Female [ref]	1	1
Male	1.08 [0.98-1.19]	
**Race**		
White [ref]	1	
Black	0.99 [0.80-1.24]	
Other	0.87 [0.66-1.15]	
**Cancer Type**		
Breast [ref]	1	1
Colorectal	1.82 [1.49-2.22]	1.54 [1.24-1.92]
Esophageal	4.02 [3.29-4.92]	2.34 [1.84-2.97]
Liver	2.74 [2.01-3.73]	1.81 [1.30-2.53]
Melanoma	1.76 [1.36-2.28]	2.29 [1.71-3.08]
Ovarian	4.68 [3.68-5.94]	2.04 [1.56-2.67]
Pancreatic	7.62 [6.39-9.09]	4.91 [3.98-6.05]
Prostate	0.21 [0.13-0.34]	0.10 [0.06-0.16]
**Stage (TNM)**		
1 [ref]	1	1
2	2.70 [1.93-3.77]	2.37 [1.68-3.32]
3	4.94 [3.53-6.91]	4.44 [3.13-6.41]
4	16.84 [12.30-23.06]	7.46 [5.32-10.45]
**Histology**		
Well differentiated [ref]	1	1
Moderately differentiated	1.11 [0.87-1.42]	2.03 [1.58-2.62]
Poorly differentiated	2.23 [1.75-2.85]	3.34 [2.59-4.31]
Undifferentiated	2.59 [1.81-3.70]	3.27 [2.24-4.78]
**Treatment type**		
Surgery	0.30 [0.27-0.33]	3.95 [0.34-0.46]
Chemotherapy	2.81 [2.51-3.14]	1.13 [0.99-1.30]
Radiation Therapy	0.60 [0.54-0.67]	0.78 [0.69-0.89]
Immunotherapy	1.19 [1.00-1.41]	0.85 [0.71-1.02]
**NLR**		
< median [ref]	1	1
> median	2.19 [1.98-2.43]	**1.43 [1.29-1.59]**

B

	Disease-Specific Survival (By Group)	5 Year Disease-Specific Survival (By Group)	10 Year Disease-Specific Survival (By Group)
	Multivariate HR [95% CI]	Multivariate HR [95% CI]	Multivariate HR [95% CI]
**Age**			
Under 60	1.53 [1.26-1.84]	1.56 [1.28-1.90]	1.54 [1.28-1.86]
60 or Over	1.42 [1.25-1.61]	1.43 [1.25-1.63]	1.42 [1.25-1.62]
**Sex**			
Female	1.38 [1.19-1.61]	1.40 [1.19-1.63]	1.43 [1.23-1.65]
Male	1.42 [1.23-1.64]	1.43 [1.23-1.66]	1.38 [1.19-1.61]
**Race**			
White	1.44 [1.29-1.61]	1.47 [1.31-1.65]	1.45 [1.29-1.62]
Black	1.94 [1.20-3.15]	1.90 [1.16-3.13]	1.94 [1.19-3.15]
Other	1.42 [0.71-2.86]	1.36 [0.66-2.81]	N/A
**Cancer Type**			
Breast	1.28 [0.91-1.79]	1.22 [0.94-1.79]	1.28 [0.90-1.81]
Colorectal	1.39 [1.06-1.81]	1.46 [1.11-1.94]	1.41 [1.08-1.83]
Esophageal	1.41 [1.08-1.83]	1.42 [1.09-1.85]	N/A
Liver	1.35 [0.77-2.38]	1.35 [0.77-2.38]	N/A
Melanoma	1.82 [1.15-2.88]	1.82 [1.10-2.99]	1.82 [1.15-2.90]
Ovarian	1.56 [1.06-2.29]	1.72 [1.15-2.58]	N/A
Pancreatic	1.61 [1.37-1.89]	1.64 [1.40-1.92]	N/A
Prostate	2.93 [0.94-9.18]	2.20 [0.67-7.24]	2.93 [0.94-9.18]
**Stage (TNM)**			
1	1.32 [0.71-2.47]	1.22 [0.58-2.55]	1.14 [0.59-2.17]
2	1.26 [0.90-1.76]	1.18 [0.83-1.69]	1.22 [0.87-1.72]
3	1.01 [0.70-1.46]	1.09 [0.72-1.64]	1.03 [0.71-1.50]
4	2.10 [1.69-2.60]	2.10 [1.68-2.62]	N/A
**Histology**			
Well differentiated	1.33 [0.82-2.15]	1.32 [0.80-2.20]	1.28 [0.79-2.08]
Moderately differentiated	1.28 [1.03-1.60]	1.29 [1.03-1.63]	1.28 [1.03-1.60]
Poorly differentiated	1.27 [1.03-1.56]	1.32 [1.06-1.64]	1.26 [1.03-1.56]
Undifferentiated	0.90 [0.45-1.82]	0.74 [0.35-1.55]	0.90 [0.44-1.84]
**Treatment type**			
Surgery only	1.35 [0.96-1.91]	1.35 [0.94-1.95]	1.39 [0.98-1.96]
Chemotherapy only	1.88 [1.54-2.30]	N/A	N/A
Radiation therapy only	2.10 [0.64-6.90]	2.10 [0.64-6.90]	2.10 [0.64-6.89]
Immunotherapy only	3.02 [0.52-17.51]	N/A	N/A

Table S1. Univariate and multivariable Cox proportional hazards models identify the adjusted hazard ratio and 95% confidence interval for NLR greater than or equal to the whole cohort median, where all covariates significant in univariate analysis are included in the multivariable model (**A**). Adjusted HRs and 95% CIs are then calculated within each patient subgroup, demonstrating the variation in strength of association between NLR and survival outcomes (disease-specific survival, five year disease-specific survival, ten year disease-specific survival) between patients with different demographic and clinical characteristics (**B**).

S1e. Optimal cutoffs

	Subgroup median	Optimal cutpoint
All	2.74	3.33
Under 60	2.51	3.10
60 or over	2.86	3.27
Female	2.57	3.33
Male	2.88	3.19
White	2.79	3.27
Black	2.08	2.13
Other	2.40	3.00
Breast	2.14	2.88
Colorectal	2.87	3.41
Esophageal	3.05	3.27
Liver	3.11	2.45
Melanoma	2.62	2.62
Ovarian	4.30	4.76
Pancreatic	3.13	3.24
Prostate	2.32	3.21
Stage I	2.18	3.40
Stage II	2.39	2.29
Stage III	2.70	3.56
Stage IV	2.75	3.29
Well differentiated	2.44	2.28
Moderately differentiated	2.50	3.09
Poorly differentiated	2.78	3.33
Undifferentiated	3.30	3.76
Surgery only	2.45	3.09
Chemotherapy only	3.72	3.64
Radiation only	2.41	3.20
Immunotherapy only	2.75	2.78

Table S2. Cutoffs for high NLR within each patient subgroup, calculated using both the group-specific median and the maximal log-rank test statistic method for identifying optimal thresholds for survival analysis within a continuous variable.

	OS (By Group)	5 Year OS (By Group)	10 Year OS (By Group)
	Multivariate HR [95% CI]	Multivariate HR [95% CI]	Multivariate HR [95% CI]
**Age**			
Under 60	1.72 [1.49-1.99]	1.84 [1.57-2.14]	1.74 [1.50-2.01]
60 or Over	1.79 [1.64-1.96]	1.85 [1.68-2.04]	1.82 [1.66-1.99]
**Sex**			
Female	1.77 [1.59-1.98]	1.93 [1.71-2.18]	1.82 [1.63-2.05]
Male	1.75 [1.57-1.94]	1.74 [1.56-1.95]	1.74 [1.57-1.94]
**Race**			
White	1.76 [1.63-1.91]	1.83 [1.68-1.99]	1.79 [1.65-1.94]
Black	1.99 [1.38-2.87]	1.98 [1.36-2.90]	1.97 [1.36-2.86]
Other	1.82 [1.12-2.95]	1.96 [1.19-3.25]	N/A
**Cancer Type**			
Breast	1.63 [1.30-2.04]	1.72 [1.31-2.27]	1.69 [1.33-2.15]
Colorectal	1.69 [1.40-2.03]	1.80 [1.46-2.21]	1.67 [1.38-2.02]
Esophageal	1.77 [1.45-2.16]	1.75 [1.42-2.15]	N/A
Liver	1.93 [1.41-2.63]	2.06 [1.49-2.85]	N/A
Melanoma	1.74 [1.27-2.38]	2.03 [1.38-2.98]	2.03 [1.45-2.84]
Ovarian	2.19 [1.56-3.10]	2.28 [1.59-3.26]	N/A
Pancreatic	1.79 [1.57-2.04]	1.83 [1.60-2.08]	N/A
Prostate	2.19 [1.48-3.25]	2.48 [1.54-4.00]	2.21 [1.46-3.33]
**Stage (TNM)**			
1	1.98 [1.43-2.74]	2.23 [1.46-3.38]	1.99 [1.39-2.84]
2	1.48 [1.18-1.89]	1.63 [1.23-2.17]	1.39 [0.98-1.97]
3	1.46 [1.10-1.93]	1.69 [1.23-2.31]	1.52 [1.14-2.02]
4	2.38 [1.96-2.88]	2.45 [2.01-2.99]	N/A
**Histology**			
Well differentiated	1.57 [1.16-2.14]	1.61 [1.14-2.28]	1.55 [1.12-2.13]
Moderately differentiated	1.75 [1.52-2.03]	1.92 [1.63-2.26]	1.81 [1.56-2.10]
Poorly differentiated	1.68 [1.43-1.97]	1.71 [1.45-2.03]	1.67 [1.42-1.96]
Undifferentiated	1.37 [0.78-2.40]	1.40 [0.78-2.51]	1.35 [0.77-2.38]
**Treatment type**			
Surgery only	1.53 [1.27-1.85]	1.74 [1.39-2.19]	1.55 [1.27-1.89]
Chemotherapy only	1.98 [1.69-2.32]	N/A	N/A
Radiation therapy only	2.67 [1.61-4.43]	3.67 [1.95-6.91]	2.96 [1.73-5.07]
Immunotherapy only	1.97 [0.50-7.79]	N/A	N/A

	DSS (By Group)	5 Year DSS (By Group)	10 Year DSS (By Group)
	Multivariate HR [95% CI]	Multivariate HR [95% CI]	Multivariate HR [95% CI]
**Age**			
Under 60	1.66 [1.38-1.98]	1.76 [1.46-2.13]	1.68 [1.40-2.02]
60 or Over	1.66 [1.47-1.88]	1.69 [1.49-1.92]	1.66 [1.47-1.88]
**Sex**			
Female	1.77 [1.53-2.06]	1.83 [1.57-2.14]	1.79 [1.54-2.08]
Male	1.57 [1.36-1.81]	1.60 [1.38-1.85]	1.58 [1.37-1.82]
**Race**			
White	1.66 [1.49-1.85]	1.72 [1.54-1.92]	1.70 [1.51-1.87]
Black	1.92 [1.19-3.10]	1.88 [1.15-3.09]	1.92 [1.18-3.11]
Other	1.67 [0.87-3.23]	1.47 [0.75-2.90]	1.67 [0.87-3.23]
**Cancer Type**			
Breast	1.48 [1.07-2.04]	1.41 [0.98-2.04]	1.48 [1.06-2.07]
Colorectal	1.61 [1.24-2.08]	1.74 [1.32-2.28]	1.62 [1.25-2.11]
Esophageal	1.59 [1.22-2.07]	1.61 [1.23-2.11]	N/A
Liver	1.44 [0.77-2.68]	1.44 [0.77-2.68]	N/A
Melanoma	1.83 [1.16-2.90]	1.83 [1.11-3.00]	1.83 [1.15-2.91]
Ovarian	2.09 [1.40-3.13]	2.27 [1.49-3.45]	N/A
Pancreatic	1.67 [1.42-1.95]	1.69 [1.44-1.98]	N/A
Prostate	1.79 [0.65-4.98]	1.34 [0.45-3.99]	1.79 [0.65-4.98]
**Stage (TNM)**			
1	1.55 [0.76-3.16]	1.55 [0.69-3.48]	1.40 [0.67-2.94]
2	1.43 [1.01-2.01]	1.38 [0.95-2.00]	1.49 [1.17-1.90]
3	1.16 [0.80-1.68]	1.33 [0.88-2.00]	1.20 [0.82-1.75]
4	2.29 [1.83-2.88]	2.32 [1.84-2.93]	2.27 [1.81-2.85]
**Histology**			
Well differentiated	1.73 [1.03-2.88]	1.67 [0.98-2.83]	1.67 [0.98-2.78]
Moderately differentiated	1.53 [1.24-1.88]	1.60 [1.28-1.98]	1.54 [1.25-1.90]
Poorly differentiated	1.59 [1.29-1.95]	1.67 [1.35-2.06]	1.58 [1.28-1.94]
Undifferentiated	0.89 [0.44-1.79]	0.81 [0.39-1.65]	0.86 [0.42-1.77]
**Treatment type**			
Surgery only	1.36 [0.97-1.92]	1.35 [0.94-1.94]	1.43 [1.01-2.01]
Chemotherapy only	1.84 [1.51-2.25]	N/A	N/A
Radiation therapy only	3.65 [1.15-11.54]	3.65 [1.15-11.54]	3.65 [1.15-11.54]
Immunotherapy only	3.02 [0.52-17.51]	N/A	N/A

Tables S3, S4. Adjusted Cox proportional hazards models identify the adjusted hazard ratio and 95% confidence interval for the association between high NLR (greater than or equal to the optimal group cutoff as defined in **Table S3)** and overall and disease-specific survival outcomes (all-time survival, five year survival, ten year survival).
